# Tension Pneumocephalus: A Rare Complication of Transsphenoidal Resection of a Pituitary Macroadenoma

**DOI:** 10.7759/cureus.4623

**Published:** 2019-05-08

**Authors:** Shumaila M Iqbal, Aalia J Khan, Cassandra Zhi

**Affiliations:** 1 Internal Medicine, University at Buffalo / Sisters of Charity Hospital, Buffalo, USA; 2 Internal Medicine, Drexel University College of Medicine, Philadelphia, USA

**Keywords:** transsphenoidal surgery, tension pneumocephalus, pituitary adenoma

## Abstract

Tension pneumocephalus (TP) is described as the presence of a large amount of air in the cranial cavity, compressing the parenchyma and ventricles. It is a rare neurosurgical emergency and has been reported in only a handful of cases as a complication of transsphenoidal resection of a pituitary adenoma. Our reported case is an addition to the series of those cases. A 60-year-old male patient underwent transsphenoidal resection of a pituitary macroadenoma. Computed tomography (CT) of the head performed post-procedure showed post-surgical changes with no identification of any acute intracerebral processes. On postoperative Day 2, the patient had a bout of sneezing and since that time, he was noted to be more altered in terms of his mentation and lethargic with no focal neurological deficits. A repeat CT of the head showed a large amount of air in the intracranial cavity compressing the brain parenchyma with slit-like appearances of the cerebral ventricles. The patient underwent emergent bifrontal air evacuation through burr holes. A cerebrospinal fluid leak was also noted while reconstructing the skull base. A postoperative CT scan showed marked resolution of TP. The patient improved clinically, was discharged home five days later, and was monitored closely by the surgical team on an outpatient basis.

## Introduction

Pneumocephalus (PNC) is the presence of air within the intracranial cavity. It can be located in the epidural, subarachnoid, intraventricular, intracerebral, or subdural space, with the subdural space being the most frequent. When PNC causes intracranial hypertension and has a mass effect, it is called tension pneumocephalus (TP) [1]. This is a rare, but treatable, neurosurgical emergency. Trauma is the most common cause of TP. Uncommon causes of TP include cranial or spinal surgeries as well as ear, nose, and throat (ENT) surgical procedures, such as paranasal sinus surgery, nasal septum resection, or nasal polypectomy [1-2]. Clinically, TP can present as restlessness, altered mentation, focal neurological deficits, or sometimes even cardiac arrest. On imaging, TP can often give the “Mount Fuji sign,” which is air in between the frontal tips [3], or the “Peaking sign” when there is a bilateral compression of the frontal lobes without separation of the frontal tips [4]. The Mount Fuji sign indicates more severe PNC than the Peaking sign and indicates the necessity of emergent decompression. Reported below is a very rare complication of TP developing secondary to transsphenoidal resection of pituitary macroadenoma in our 60-year-old patient. The computed tomography (CT) head performed showed the Mount Fuji sign due to mass effect on brain parenchyma by intracranial air.

## Case presentation

**Figure 1:**
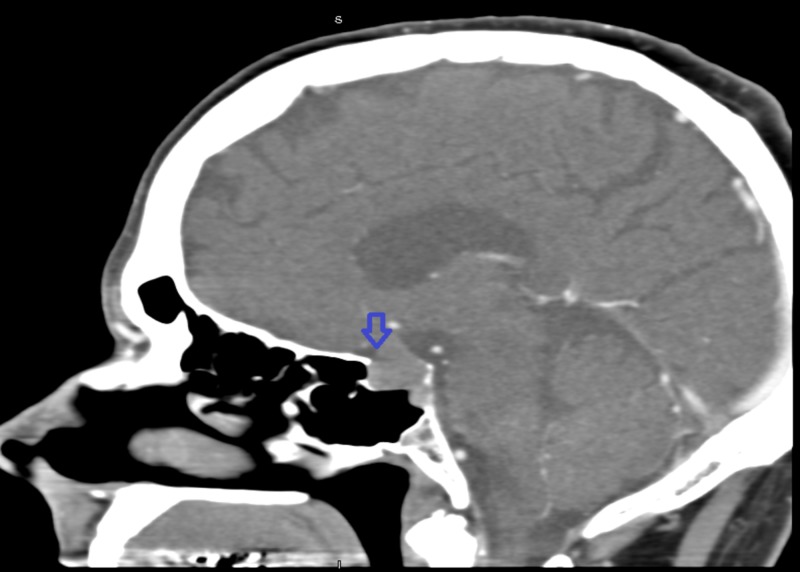
Computed tomography of the head with arrowhead pointing towards the pituitary macroadenoma

Our patient is a 60-year-old male with a past medical history of hypertension and peptic ulcer disease. He presented to the outpatient clinic of the neurosurgery department for progressively worsening headache and repeated episodes of syncope. As a part of outpatient workup, he underwent CT of the head, which showed lobulated pituitary soft tissue mass noted in the sella turcica region. The mass was 1.8 cm x 1.7 cm x 1.4 cm (macroadenoma) with displacement of the optic chiasm (Figure 1).

No sella bony erosion was identified. Visual field testing performed was reported to be normal. He was referred to an endocrinologist for further evaluation of the pituitary macroadenoma. Hormonal workup ordered by the endocrinologist revealed low testosterone 85 ng/dl (normal value: 270-1070 ng/dl) and low luteinizing hormone 1.0 mIU/ml (normal value: 1.6-15.5 mIU/ml) as the only hormonal abnormalities. The results of other hormonal workup included: plasma adrenocorticotropic hormone 13 pg/ml (normal value: 6-50 pg/ml), follicle stimulating hormone 3.4 mIU/ml (normal value: 1.6-8 mIU/ml), insulin-like growth factor-1 100 ng/ml (normal value 41-279 ng/ml), prolactin 6.2 ng/ml (normal value: 2-18 ng/ml), and thyroid stimulating hormone 3.09 mIU/l (normal value 0.40-4.50 mIU/l).

The endocrinologist referred this neurosurgery team patient further to the ENT service for surgical management of the pituitary macroadenoma. The patient underwent CT-guided stereotactic endoscopically assisted transsphenoidal resection of the pituitary macroadenoma by the ENT team with the collaboration of the neurosurgery team. During the procedure, the anterior portion of the sphenoid sinus was entered and mucosa was removed. Using the Brainlab stereotactic system (Brainlab AG, Munich, Germany), the lesion was identified posterior to the sphenoid sinus. At that point, the posterior wall of the sphenoid sinus was removed with an osteotome and a Kerrison rongeur. The posterior capsule of the pituitary was opened sharply with a number 11 blade and the pituitary tumor was then removed with a ring curet. The frozen section was consistent with a pituitary adenoma. The tumor was then removed with suction and pituitary rongeurs. Inspection with the endoscope revealed gross total resection of the adenoma. At that point, the tumor cavity was packed with Surgiflo and the posterior capsule was then lined with Duragen (Integra LifeSciences, NJ, US). The mucosal flap was then placed over the posterior wall of the sphenoid and reinforced with Duraseal (Integra LifeSciences) to provide watertight closure. The patient initially did well after the surgical procedure. He was alert and oriented. He was able to ambulate independently and was tolerating a regular diet as well. No headache, confusion, dizziness nasal congestion, vision changes (blurry vision, loss of color perception, diplopia), or cognitive dysfunction was reported by the patient postoperatively. The neurological exam also remained non-focal. Head CT performed on the postoperative day showed post-surgical procedure changes, with no acute intracerebral process that could be identified on imaging. The patient did not experience diabetes insipidus after the procedure and the serum sodium level remained within the normal range consistently. He was started on hydrocortisone 20 mg in the morning and 10 mg at dinner time after the surgery.

**Figure 2:**
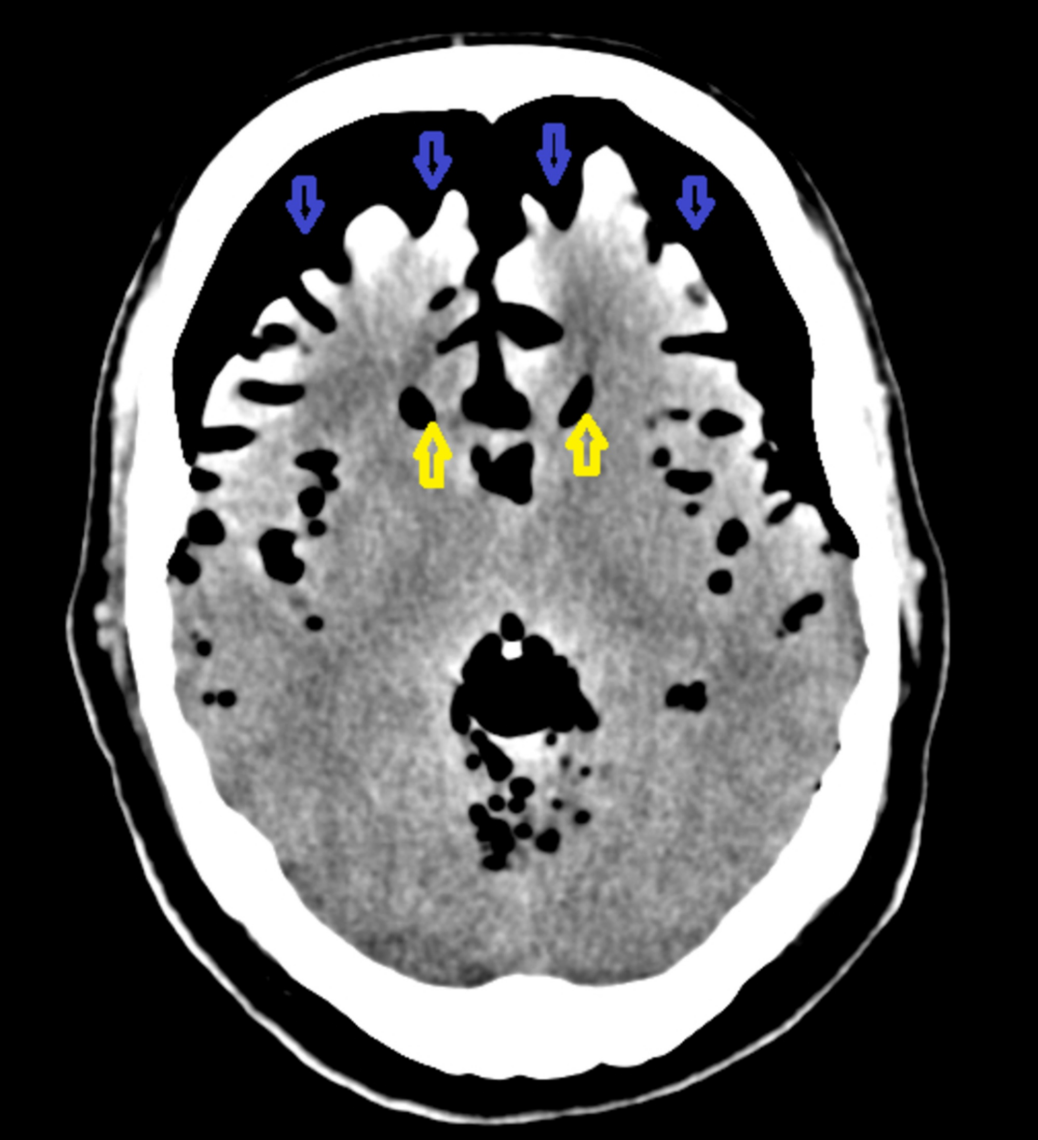
Computed tomography of the head performed on postoperative Day 2 shows the Mount Fuji sign secondary to tension pneumocephalus. The blue-colored arrowhead is pointing towards intracranial air. The yellow-colored arrowhead is pointing towards the slit-like ventricles.

On postoperative Day 2, the patient had a sneezing episode. Almost immediately after that, he had a cognitive decline and started having a generalized headache. The mental status decreased further and he became somnolent but was responsive to verbal stimulus. He was found to be oriented to self and place and was able to follow commands but his response was very slow. He mentioned feeling weak in general. The neurological exam performed remained non-focal. CT of the head was repeated, which showed extensive pneumocephalus that had not been present in the previous study. Evaluation of the brain parenchyma was limited in this study due to artifacts from the large amount of pneumocephalus. However, there was a mass effect on the brain parenchyma due to air and the ventricular system appeared to be decompressed and slitlike (Figure 2).

**Figure 3:**
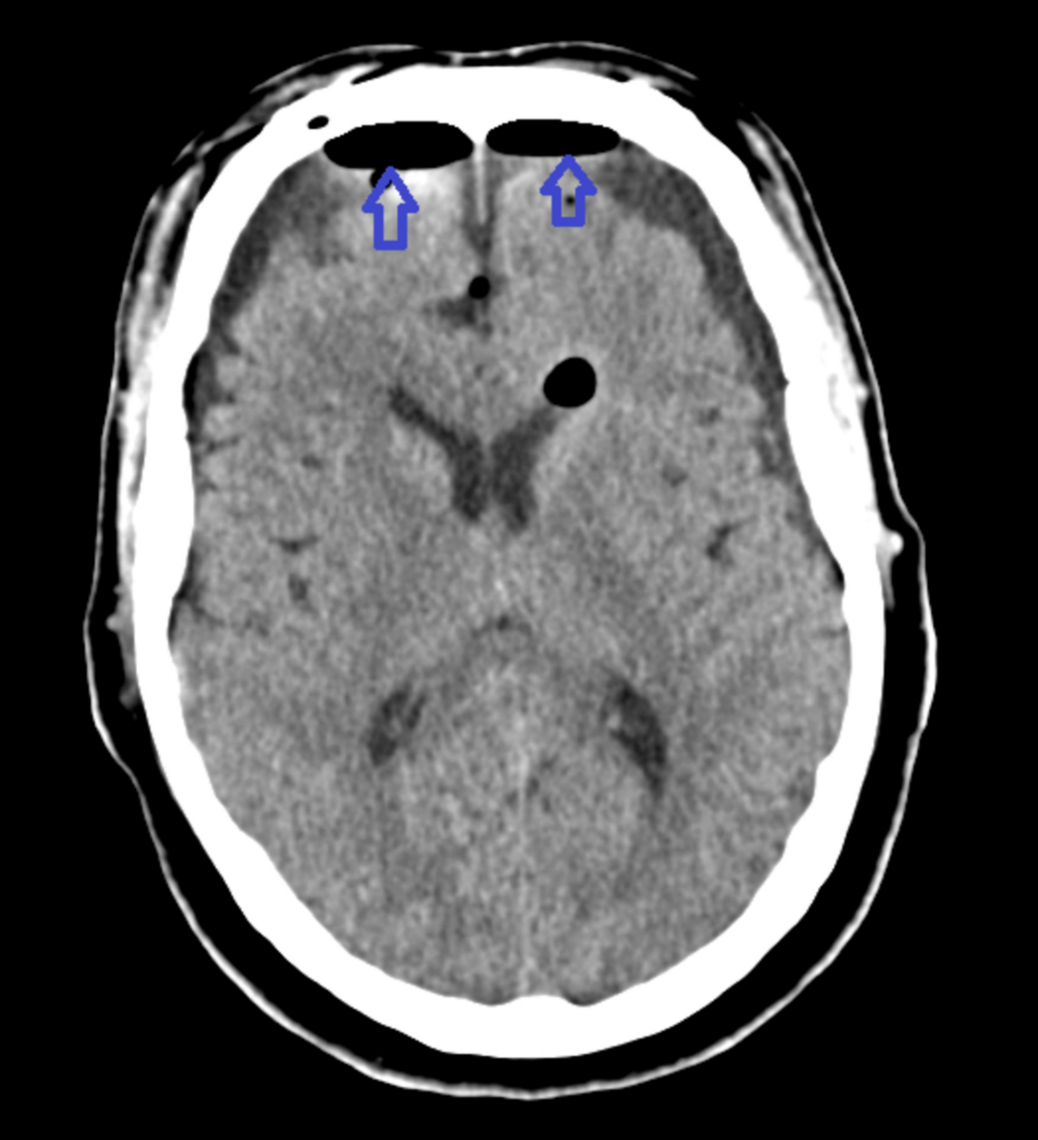
Computed tomography of the head showing significant improvement in tension pneumocephalus post-procedure The blue-colored arrowheads point towards small bifrontal collections, likely hygromas.

The likely suspicion was dislodgment of the skull base repair at the time of the initial surgery, causing TP to the patient. The patient was taken back to the operative room immediately. An urgent bifrontal burr hole evacuation of intracranial tension pneumocephalus was performed. The bilateral nasal septal flaps used previously to reconstruct the skull base were found to be intact, with no visible dehiscence or displacement from the skull base based on magnified endoscopic examination. The Duraseal was suctioned and the flaps were taken down. Duragen was then placed within the pituitary fossa. This was removed gently with suction. There was a cerebrospinal fluid leak (CSF) noted in the pituitary fossa. Duragen wrapped in Surgicel (Ethicon, Inc., NJ, US) was then placed back into the pituitary fossa as well as the inferior aspect of the sella and then the nasoseptal flaps reapproximated, providing an excellent seal of the entire posterior superior wall of the sphenoid sinus, including the openings into the sella. Additional Duraseal was placed and the flap was supported by two 8 cm Nasopores ((Stryker, MI, US): one inferior to the flap to provide support and prevent inferior displacement of the flap and one anterior to the flaps to compress the flaps against the opening into the sella. Two additional Nasopores, as well as surgifoam, were placed within the sinonasal cavity, one on either side for hemostatic control. Repeated CT scan performed post-procedure showed significant improvement in pneumocephalus, with small bifrontal collections, likely hygromas (Figure 3).

The patient improved clinically post-procedure and had an improvement in cognition and mental functional status. He was started on intravenous (IV) vancomycin 0.75 gm daily and IV cefepime 2 gm every eight hours for a total of five days in duration. For seizure prophylaxis, the patient was started on IV Keppra 1000 mg twice a day and switched to oral Keppra 1000 mg twice a day at the time of discharge. The patient’s symptoms improved post-procedure and cognition returned to normalization. On postoperative Day Five, the patient was discharged home with recommended outpatient endocrinology, ENT, and neurosurgery follow-up.

## Discussion

The term TP was first described by Ectors, Kessler, and Stern in the year 1962 as PNC causing a mass effect to the brain parenchyma [5]. The mechanism of development of PNC can be described by two possible theories [6]. The first one is the Dandy theory of ‘ball valve,’ which is described as the unidirectional flow of air from outside into the cranial cavity, where the air remains trapped and cannot exit [7]. The second proposed theory is Horowitz’s ‘inverted-soda-bottle effect,’ which described the generation of negative intracranial pressure (ICP) occurring as a result of excessive CSF loss due to numerous methods. For example, drainage in a physiological way during the Valsalva maneuver or through the therapeutic lumbar drain could cause this effect [8]. Our patient’s symptom of altered mental status (AMS) and lethargy started to happen right after a bout of sneezing, which happened the morning following a transsphenoidal surgical resection of pituitary adenoma. As per evidence-based literature, sneezing is similar to the Valsalva maneuver, which increases the intrathoracic pressure [9]. This is associated with a simultaneous increase in ICP [9]. The same phenomenon might have contributed towards the CSF leak and TP development in our patient because the CSF leak was also noted during the surgical correction of TP.

The onset of air accumulation into the intracranial cavity after cranial surgeries can be acute (<72 two hours) or delayed (>72 hours). A comprehensive literature review by Pillai et al. on 18 patients with TP reports delayed onset in most of the cases [2]. However, there was also a reported case for the development of PNC three weeks post-surgery in a 49-year-old patient who had transsphenoidal surgery for a pituitary tumor [10]. In our patient, the onset can be considered acute, as it happened the very next day after the first transsphenoidal surgical resection for pituitary macroadenoma and PNC progressed very rapidly to symptomatic TP.

TP can be a life-threatening neurosurgical emergency if not treated in a timely manner and can have devastating outcomes. Its presence should be considered highly suspicious whenever a sudden onset of altered mentation or lethargy is noted in a patient who had undergone a recent transsphenoidal surgical procedure or other forms of craniotomies. Prompt evaluation by magnetic resonance imaging (MRI) or CT imaging can help make a timely diagnosis, which would lead towards surgical correction for TP. The surgical correction for TP includes emergent intracranial air evacuation through burr holes along with the surgical repair of the causative factor. Frequent follow-ups with imaging studies may help track the redevelopment of PNC after the surgical correction of TP [2].

## Conclusions

TP is a rare, but very serious, complication of transsphenoidal resection of a pituitary macroadenoma. It should be considered in any patient who develops altered mental status, lethargy, restlessness, or focal neurological deficit acutely or sub-acutely after surgical resection of a pituitary tumor. Diagnostic imaging should not be delayed for highly suspicious TP. If not corrected via surgery emergently, unrecognized and untreated TP can have devastating neurological outcomes.
